# Development and Validity Study of a Mobile Corsi Block Test for Visuospatial Working Memory in Community-Dwelling Older Adults

**DOI:** 10.3390/brainsci16080854

**Published:** 2026-08-13

**Authors:** Dahye Ham, Eunyoung Cho, Sungwon Choi

**Affiliations:** 1 Department of Psychology, Duksung Women’s University, Samyang-ro, Dobong-gu, Seoul 01369, Republic of Korea; dahye8305@gmail.com; 2 Department of Rehabilitation Medicine, CHA Bundang Medical Center, School of Medicine, CHA University, Seongnam 13497, Republic of Korea; a220242@chamc.co.kr

**Keywords:** visuospatial working memory, Corsi block-tapping test, mobile assessment, older adults

## Abstract

**Highlights:**

**What are the main findings?**

- The mobile Corsi Block Tapping Test demonstrated significant correlations with the Working Memory Test of the Comprehensive Attention Test, supporting its concurrent validity.
- Significant correlations were also observed between The Mobile Corsi Block Tapping Test and established standardized measures, including the Korean Type Memory Test Type B and the Snellgrove Maze Task, supporting its convergent validity.

**What are the implications of the main findings?**

- The Mobile Corsi Block Tapping Test developed in this study demonstrated validity as a measure of visuospatial working memory in older adults.
- Its mobile-based format may provide a more accessible approach for assessing visuospatial working memory in older adults.

**Abstract:**

**Background/Objectives**: The Corsi Block Tapping Test (CBTT) is a widely used measure of visuospatial working memory; however, its traditional paper-and-pencil administration is limited by inter-examiner variability in procedure and restricted accessibility. To address these limitations, the present study developed a Korean mobile version of the CBTT and examined its reliability and validity among community-dwelling older adults. **Methods**: The CBTT was administered to 120 Community-Dwelling older adults. Participants were aged 65 years or older, had no neuropsychiatric disorders or visual impairments, and met the cognitive and depression screening criteria. The CBTT was compared with established standardized measures, including the Spatial Working Memory Test (SWMT), the Korean-Trail Making Test for the Elderly Type B (K-TMT-e Type B), and the Snellgrove Maze Test (SMT). **Results**: The internal consistency of the CBTT was acceptable (α = 0.767). The backward metrics, particularly the CBTT Backward Correct Responses, showed consistent significant correlations with all three criterion measures, whereas the forward metrics were largely non-significant. Correlation magnitudes were generally small to moderate. **Conclusions**: These findings provide preliminary evidence supporting the validity of the CBTT, particularly its backward metrics, as a measure of visuospatial working memory in older adults. However, the CBTT should currently be regarded as a preliminary assessment tool rather than a standalone diagnostic or screening instrument.

## 1. Introduction

Cognitive function plays a crucial role in enabling individuals to carry out daily life activities [1], and among its components, memory serves as the foundation for various cognitive functions, including decision-making [2]. In particular, working memory is a higher-order cognitive system that involves temporarily storing information while simultaneously manipulating it, representing a core cognitive domain for navigating complex daily activities [3]. Numerous studies have shown that working memory capacity declines with advancing age [4]. This age-related decline in working memory is more pronounced in complex tasks that require simultaneous storage and processing, compared to simple short-term memory tasks that demand less conscious effort [5].

When working memory declines, individuals may experience difficulties in daily life, such as misplacing objects or getting lost. Such difficulties are particularly associated with visuospatial working memory, which refers to the temporary storage and manipulation of visual and spatial information [6]. Demands on visuospatial working memory arise frequently in everyday life. For instance, the ability to find one’s way and navigate to familiar locations relies heavily on visuospatial working memory, and a decline in this ability can negatively affect independent living and safety [7,8]. Therefore, early detection of visuospatial working memory decline and timely intervention to delay its progression have been emphasized, highlighting the need for the development of assessment tools that can rapidly and conveniently measure visuospatial working memory.

The Corsi block-tapping task is a nonverbal, visuospatial task that assesses visuospatial working memory [9,10]. Because this task requires minimal verbal ability, it can selectively measure visuospatial domains while minimizing the confounding influence of language impairment on task performance [11]. Furthermore, as a standardized measure with established reliability and validity, it has been widely used across populations ranging from preschool children to older adults. Despite its utility, however, the traditional Corsi block-tapping task has several limitations. First, its administration and scoring procedures are not sufficiently standardized [12]. For example, examiner-related variability—such as a gradual slowing in the pace of tapping blocks as the test progresses, or the use of a pencil rather than a finger—may influence examinee performance [13,14]. Second, because the task must be administered in person by a trained examiner at a designated facility, it incurs time and financial costs, thereby limiting accessibility. Third, although examiners can manually measure reaction time, precise measurement is difficult and inter-rater error may occur, limiting the ability to systematically and accurately capture detailed behavioral indices. Given that such detailed indices may provide a more nuanced understanding of an examinee’s underlying cognitive processes [2,13], measuring them accurately may help enhance the sensitivity and diagnostic utility of the task.

To address these limitations of the traditional Corsi block-tapping task, a computerized version was developed. In the computerized version, administration and scoring procedures were standardized to minimize examiner-related variability in test procedures, enabling precise stimulus presentation, accurate reaction time measurement, and automated scoring [13,15]. Furthermore, this format allows for the precise collection of micro-behavioral indices such as reaction time and offers greater flexibility with respect to time and location. Such precisely collected, standardized data may help skilled neuropsychologists interpret and evaluate an examinee’s cognitive functioning more thoroughly.

The computerized Corsi block-tapping task can be implemented on a variety of devices, including computers [15], tablets [16], and smartphones. While computer- or tablet-based assessments require a dedicated device to be prepared for administration, smartphones offer higher accessibility, as most individuals already possess and use them in daily life [17]. This characteristic enables participation in the assessment without the need for additional equipment or travel, further enhancing temporal and economic accessibility. In particular, for community-dwelling older adults who may face barriers to visiting healthcare facilities, an assessment utilizing their own smartphone may serve as a practical alternative.

However, existing computerized versions of the Corsi block-tapping task have primarily been developed and validated in Western countries [13,18], and to date, no smartphone-based version developed and validated among older adults in Korea has been identified. Accordingly, the present study aimed to develop a Korean mobile version of the Corsi block-tapping test (CBTT) and to examine its reliability and validity among older adults. Here, “mobile” refers to a format implemented on a smartphone that individuals routinely carry in daily life.

## 2. Materials and Methods

### 2.1. Participants

This study was conducted as data of the 2019 National Life Safety Emergency Response Research Project “Development of Driving Ability Evaluation System and Mobile Application to Improve Road Safety for Elderly Drivers.”. Participants were recruited from older adults residing in Wonju-si, Gangwon-do, who voluntarily expressed interest in participating after seeing a recruitment announcement. Social and mental health variables were collected and neuropsychological assessments were conducted for about two months from June to August 2021 a total of 150 older adults. The sample size was determined based on the rule of thumb provided in the scoring criteria of the COSMIN checklist, a consensus-based standard for the selection of health measurement instruments [19]. According to this criterion, a sample size of 100 is considered “excellent,” and the sample in the present study (*N* = 150) meets this benchmark.

The following selection criteria were applied to select older adults without physical and mental health problems and cognitively non-demented. (1) Age 65 years or older; (2) No history of neuropsychiatric disease; (3) No visual impairment or color vision deficiency; (4) a T-score of 30 or higher on the Korean Mini-Mental Status Examination-2:Standard Version (K-MMSE-2:SV); (5) a score of less than 1 on the Clinical Dementia Rating (CDR); (6) a score of less than 10 on the Short Form of Geriatric Depression Scale (SGDS).

**Figure 1:**
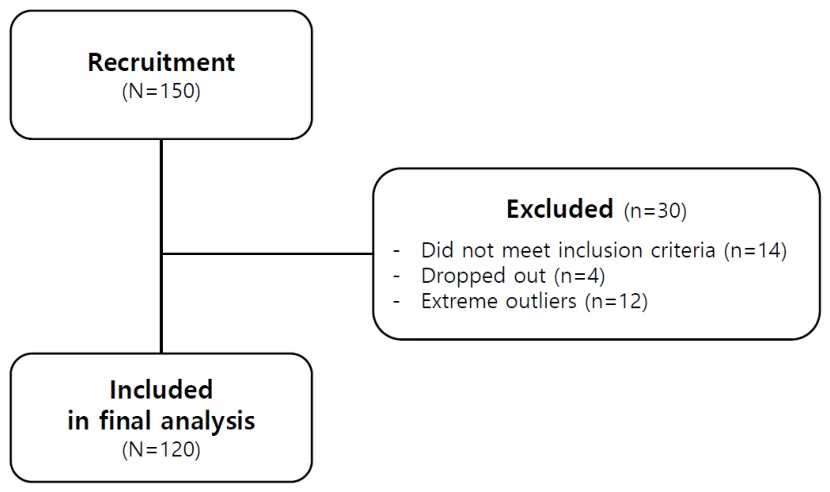
Recruitment flowchart.

14 participants who did not meet the inclusion criteria, 4 who did not complete the assessment, and 12 who exhibited extreme outliers in task performance were excluded. Outliers were identified using the interquartile range (IQR) based on the Forward Correct Responses and Backward Correct Responses of the Corsi Block-Tapping Test (CBTT) [20]. Values falling below Q1 − 1.5 × IQR or above Q3 + 1.5 × IQR were defined as outliers (see Section 2.4.5. Metrics for the rationale for variable selection), and participants identified as outliers on either of these two indices were excluded from the analysis. As a result, a total of 30 participants were excluded, yielding a final sample of 120 for analysis. The recruitment flowchart is presented in Figure 1. The demographic characteristics of the study participants are presented in Table 1. This study was reviewed and approved by the Institutional Review Board of Yonsei University Wonju Severance Christian Hospital (CR321016). The study participants were informed of the study description and confidentiality and signed an informed consent form.

### 2.2. Measurement Tools

#### 2.2.1. Korean Mini-Mental Status Examination-2:Standard Version (K-MMSE-2:SV)

The Mini-Mental State Examination-2 (MMSE-2) is a cognitive screening test originally developed by Folstein et al. [21]. In this study, we used the Korean version standardized by Kang et al. [22]. It consists of 19 items, and the subscales include time span (5 points), place span (5 points), memory registration (3 points), attention and calculation (5 points), memory recall (3 points), language (8 points), and visual organization (1 point). The minimum score is 0 and the maximum is 30, with higher scores indicating better preservation of cognitive function. T-scores (M = 50, SD = 10) are calculated based on normative samples matched for age and education, and a T-score below 30 (i.e., more than 2 standard deviations below the normative mean) is considered indicative of cognitive impairment. The test takes about 5 to 10 min.

#### 2.2.2. Clinical Dementia Rating (CDR)

The Clinical Dementia Rating (CDR) is a semi-structured interview tool that assesses six domains (memory, executive function, judgment and problem solving, social activities, home life and hobbies, hygiene and grooming) to assess the clinical stage of dementia [23]. Each of the six domains is scored on a scale of 0, 0.5, 1, 2, 3, 4, or 5, and the final CDR score is calculated by considering the scores of all domains. The calculated score is presented in two metrics: Sum of Boxes (CDR-SB) and CDR Score (Global Score), which are the sum of all six domains and the overall CDR score based on the memory test, respectively. CDR 0 means no dementia, CDR 0.5 means probable dementia, CDR 1 means mild, CDR 2 means moderate, CDR 3 means severe, CDR 4 means very severe, and CDR 5 means end-stage dementia.

#### 2.2.3. Short Form of Geriatric Depression Scale (SGDS)

The Short Form of Geriatric Depression Scale (SGDS) is a screening test for geriatric depression developed by Yesavage et al. [24] and shortened to only 15 questions by Sheikh and Yesavage [25]. In this study, we used the shortened version of the Geriatric Depression Scale, which was translated and standardized by Cho et al. [26]. Compared to other existing depression scales, it has the advantages of being easy to understand and easy to administer to the elderly in a short time. It is a 15-item dichotomous scale in which the elderly answer “yes/no” to each question. The minimum score is 0 and the maximum is 15, with higher scores indicating more severe depression. On the distribution of depression scores, a score of 5 or less is normal, 6 to 9 is moderate depressive symptoms, and 10 or more is depression [27].

### 2.3. Procedures

Prior to beginning the tasks, all participants received an explanation of the study’s purpose, tasks, and procedures from the examiner based on standardized written materials. The experiment began once participants confirmed that they had sufficiently understood the tasks. Task administration took place in a private room shielded from external stimuli, with participants completing the tasks independently without the examiner present. If desired, participants were allowed to take a break after fully completing each task before proceeding to the next.

### 2.4. Experimental Tools

This test was developed by referring to previous studies, and Sight Insight Inc. programmed the instructions related to the development principles and built a server to collect data on the subjects’ performance.

#### 2.4.1. Mobile Devices Used

**Figure 2:**
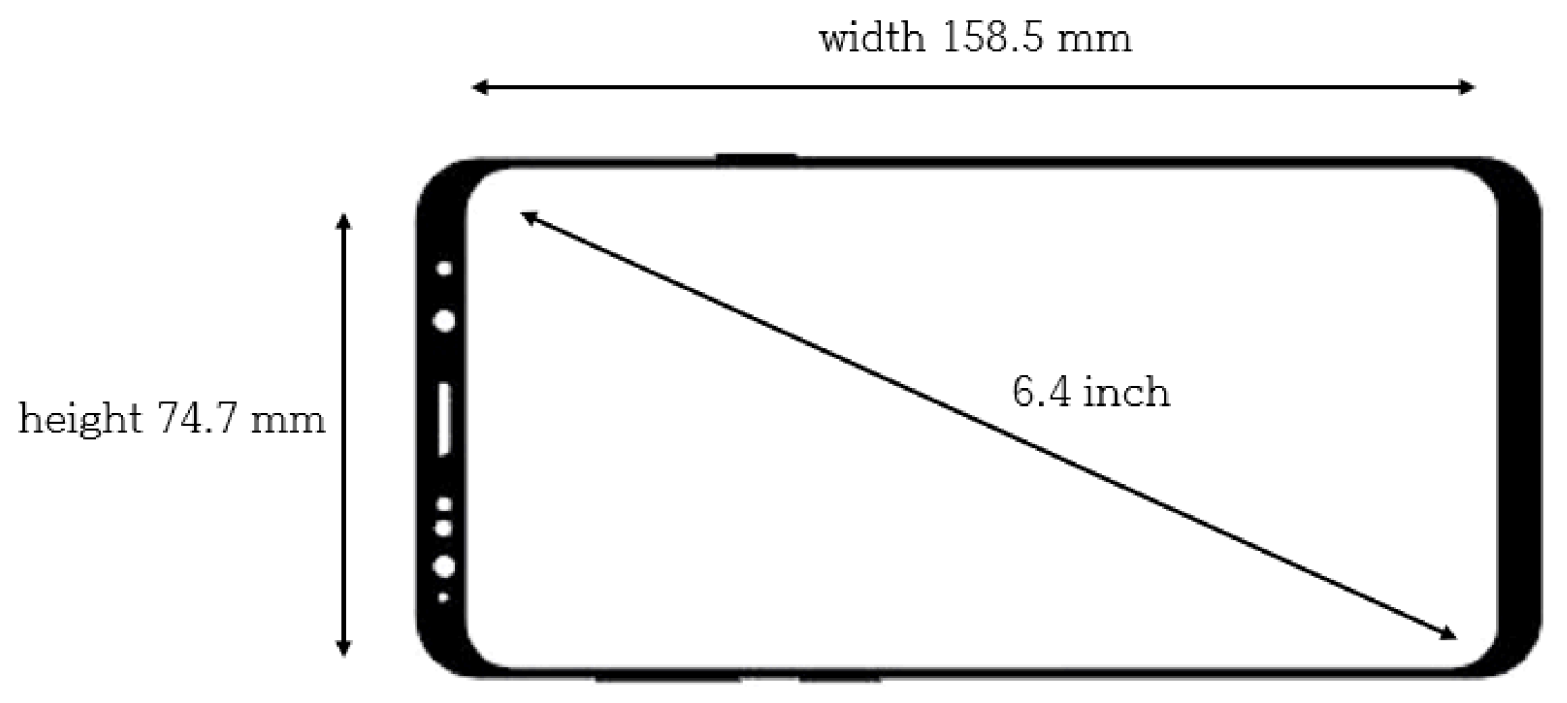
Image of the mobile device used.

In this study, the CBTT was developed for the Android operating system, considering that Android-based smartphones are widely used in Korea, particularly among older adults, who constitute the primary target population of this study (https://www.gallup.co.kr/gallupdb/reportContent.asp?seqNo=1566, accessed on 7 August 2026). The device used was the Samsung Galaxy A50 (SM-A505; Samsung Electronics, Suwon, South Korea), with dimensions of 158.5 × 74.7 × 7.7 mm (width × height × thickness) and a 6.4-inch main display with a 19.5:9 aspect ratio. The main screen is Super AMOLED and has a resolution of 2340 × 1080 (FHD+). A representative image of the device is shown in Figure 2. In order to provide similar stimuli regardless of the size of the user’s mobile device screen, we used the concept of density-independent pixel (DIP). DIP is used to ensure that the stimuli displayed on the screen are optimized for the screen size and pixel density to provide the ability to make applications compatible across all screens [28,29]. Accordingly, if the application is commercialized in the future, it is expected to be operable on a variety of Android devices beyond the one used in this study. The mobile devices used in this study were connected by a wireless network, and calls and texts were blocked while using the application to control for stimuli that could interfere with the experiment. The subjects’ responses (number of correct responses, spatial width) were automatically stored on a web server.

#### 2.4.2. Implementation Conditions

The CBTT was administered in two conditions: forward and backward. The order of blocks presented on the screen (sequence) was a standard sequence with two trials of the same sequence length [12]. Previous studies have shown that visuospatial working memory performance can vary depending on how a sequence is configured, including factors such as overall path length and the number of path crossings [14,30]. As such, sequence configuration is an important variable that may influence task outcomes. When subjects are presented with a different sequence in each trial, variations in difficulty could act as an extraneous variable affecting performance. Therefore, we used standardized sequences to control for extraneous variables. The maximum sequence length was set at 9 blocks for the forward condition and 8 blocks for the backward condition, with two different sequences of equal length presented at each level.

#### 2.4.3. Test Stimuli

**Figure 3:**
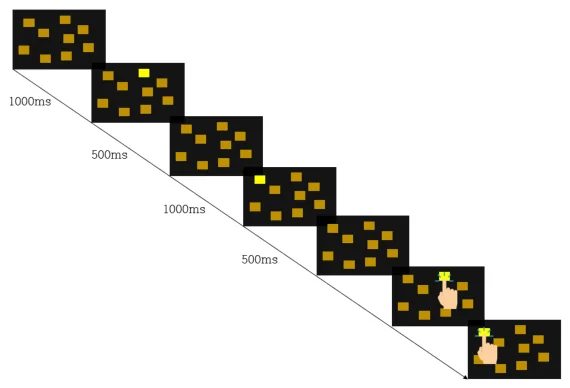
Test stimulus images from the CBTT (e.g., forward condition, sequence length 2).

**Figure 4:**
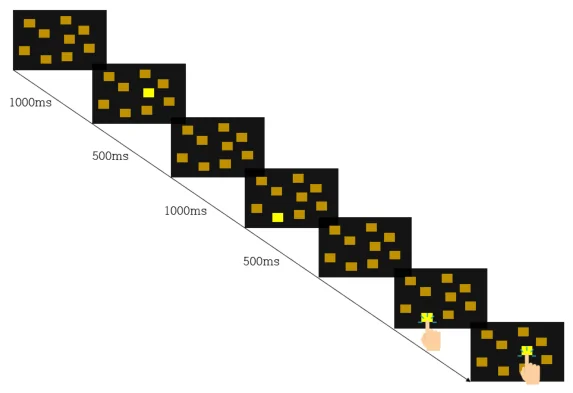
Test stimulus images from the CBTT (e.g., backward condition, sequence length 2).

We used the standard block layout proposed by Kessels et al. [12] and modified the test stimuli for the mobile version. The standard block layout consists of nine blocks numbered 1 through 9, positioned at fixed locations. The desktop is black, and the block color changes from dark yellow to light yellow only when the block is presented. The presentation time of the blocks was set to 500 ms and the inter-stimulus interval (IOI) was set to 1000 ms. The test stimuli used in this study are shown in Figure 3 and Figure 4.

#### 2.4.4. Task Administration Procedure

① Practice Trials

An auditory cue (“beep”) was presented before and after the block stimuli in each sequence to signal the start of the task to the participant. During practice trials, an auditory cue indicating a correct response (“ding-dong-dang”) was presented for correct responses, and an auditory cue indicating an incorrect response (“buzz”) was presented for incorrect responses. If the participant did not respond within a set time limit, the task automatically proceeded to the next item. If the participant did not sufficiently understand the task during the practice trials, the practice trials were repeated; once adequate understanding was confirmed, the main trials began.

② Main Trials

As in the practice trials, an auditory cue (“beep”) was presented before and after the block stimuli in each sequence to signal the start of the task. Unlike the practice trials, no auditory feedback indicating correct or incorrect responses was provided during the main trials. If the participant did not respond within a set time limit, the task automatically proceeded to the next item. The forward condition was administered first, followed by the backward condition, with the entire procedure taking approximately 3 to 5 min.

#### 2.4.5. Metrics

In this study, we measured span and correct responses for both the forward and backward conditions. Span is the length of the longest sequence among the correct trials. The number of correct responses refers to the total number of points scored, with one point for each correct sequence. According to the scoring method described by Kessels et al., the span advances to the next level if a participant succeeds on at least one of two trials at each sequence length. Given this scoring method, it suggests that participants with the same span score may exhibit different underlying performance patterns, indicating that span scores may be overestimated by chance. On the other hand, the number of correct responses can compensate for the limitations of the span because it reflects the scores of all trials. Therefore, we consider both metrics in this study.

#### 2.4.6. Criterion Measures

① The Comprehensive Attention Test (CAT)–Spatial Working Memory Test (SWMT)

The Comprehensive Attention Test (CAT) is a computer-based assessment developed and standardized to comprehensively evaluate various types of attention [31]. It consists of six subtests: visual and auditory simple selective attention, sustained attention with inhibitory control, interference selective attention, divided attention, and working memory. Among these, the present study used the Spatial Working Memory Test (SWMT) subtest to examine its association with visuospatial working memory.

The Spatial Working Memory Test (SWMT) assesses the ability to accurately remember the sequence of visually presented stimuli in either the forward or backward direction. Participants are required to memorize the order in which boxes light up on the screen and then click the boxes in the same order or in reverse order using a mouse. Through this process of remembering and processing the sequence of presented stimuli, the task measures visuospatial working memory.

The administration conditions of the SWMT consist of forward and backward conditions, similar to the CBTT. Sequences are presented in a fixed order corresponding to the type (Type A or Type B) selected before the test begins, starting with two boxes, with two trials administered at each sequence length. If the participant responds correctly on at least one of the two trials, the number of boxes presented increases by one; if both trials are answered incorrectly, the test is terminated. The maximum sequence length is 10 boxes for both the forward and backward conditions.

The stimulus presentation duration (the time from an empty box to its transition to a white box) was 1000 ms, and the inter-onset interval (IOI) between stimuli was 2000 ms. The outcome measures were the number of correct responses and span, referring to the total score and the maximum number of boxes correctly recalled, respectively. The test takes approximately 3 to 5 min to complete.

② The Korean Trail Making Test for the Elderly Type B (K-TMT-e Type B)

The Korean-Trail Making Test for the Elderly (K-TMT-e) is a paper-and-pencil test that assesses various cognitive domains, including visual search, short-term memory, working memory, and attention shifting. Owing to its simple administration and low time and cost burden, it has been widely used in both clinical and research settings. The test consists of Part A and Part B, each comprising 15 circles randomly distributed on an A4-sized sheet. In Part A, participants connect circles numbered 1 to 15 in sequential order (e.g., 1 → 2 → 3), whereas in Part B, participants alternate between circles numbered 1 to 8 and circles labeled with the days of the week, from “Monday” to “Sunday” (e.g., 1 → Mon → 2 → Tue). Participants are instructed to complete the task as quickly as possible, and if an error occurs, the examiner immediately points it out, and the participant returns to the point preceding the error to continue the task. Scoring is based on reaction time and the number of errors [32].

Part A primarily requires visual search, sustained attention, and psychomotor speed, whereas Part B involves more complex cognitive processing, including alternating attention, cognitive flexibility, working memory, visuospatial ability, and executive function. This difference arises because, unlike Part A, Part B additionally requires the ability to switch between numbers and letters, which relies on the activation of working memory. Accordingly, the present study selected Part B as the criterion measure to examine its association with visuospatial working memory.

③ The Snellgrove Maze Test (SMT)

The Snellgrove Maze Test (SMT) was originally developed to screen for safe driving competence in older drivers and assesses attention, visuoconstructional ability, and executive functions related to planning and foresight [33]. The task requires participants to trace a path from the starting point to the endpoint of a given maze as quickly and accurately as possible. It consists of a practice trial and a set of main trials, in which participants complete five mazes of varying difficulty in succession. The outcome measures are reaction time and total error count, with the latter calculated as the sum of planning errors (reaching a dead end) and wall-crossing errors (crossing the maze boundary).

### 2.5. Statistical Analysis

IBM SPSS Statistics 21.0 (IBM Corporation., Armonk, NY, USA) was used for the statistical analysis of this study. Statistical significance was defined as *p* < 0.05, and the following statistical analysis methods were used.

First, descriptive statistics (frequency analysis, means, and standard deviations) of demographic characteristics and key variables were conducted to identify the characteristics of the study participants.

Second, outliers were identified using the interquartile range (IQR) based on the forward and backward correct responses of the CBTT. To assess the stability of the main findings with respect to outlier exclusion, the same analyses were additionally conducted on the dataset including the outliers.

Third, to assess reliability, Cronbach’s α, an index of internal consistency, was calculated.

Fourth, a Spearman correlation analysis was conducted with SWMT, which is a representative working memory test among standardized tests, to verify its concurrent validity.

Firth, a Spearman correlation analysis was conducted with K-TMT-e Type B and SMT, which are representative working memory tests among standardized tests, to verify convergent validity.

## 3. Results

### 3.1. Descriptive Statistics of Demographic Characteristics and Key Variables

The descriptive statistics of the main variables are shown in Table 2. The mean CBTT forward correct responses and forward span were 7.17 (SD = 1.96) and 5.16 (SD = 1.17), respectively, and the mean backward correct responses and backward span were 6.44 (SD = 1.90) and 4.88 (SD = 1.08), respectively, for participants in this study. The mean of the SWMT forward correct responses and forward span were 4.61 (SD = 1.34) and 6.17 (SD = 2.21), respectively, and the mean of the SWMT backward correct responses and backward span were 4.94 (SD = 1.62) and 6.52 (SD = 2.56), respectively.

### 3.2. Reliability of CBTT

The internal consistency of the CBTT was estimated using Cronbach’s α, with the four indices—forward correct responses, forward span, backward correct responses, and backward span—entered as items. Cronbach’s α values of 0.70 or higher are generally considered acceptable [34], and the present study yielded α = 0.767, suggesting that the four indices demonstrate a reasonably consistent internal structure.

### 3.3. Validation of CBTT

To examine whether the CBTT validly measures visuospatial working memory, concurrent validity was assessed through its correlation with the SWMT, and convergent validity was assessed through its correlations with the K-TMT-e Type B and SMT. Shapiro–Wilk tests of normality indicated that all four measures, including the CBTT, significantly deviated from a normal distribution; therefore, Spearman correlation analyses were conducted.

#### 3.3.1. Concurrent Validity

The concurrent validity was analyzed to verify the validity of the CBTT developed in this study. The concurrent validity is a method of verifying the validity through correlation analysis with existing tests that have been guaranteed validity in order to analyze the validity of the newly produced test. The newly developed test has a high correlation with the reference test and can be considered a valuable test if it is more valid or econom ical than using the reference test. To this end, a correlation analysis was performed between CBTT and SWMT, a standardized test.

##### Correlation Analysis of CBTT and SWMT Performance

To examine the validity of the CBTT, we conducted a correlation analysis with the SWMT, a standardized test. The results are presented in Table 3. The forward correct responses correlation coefficient of CBTT and SWMT (r = 0.258, *p* < 0.01) showed a positive correlation. Forward span of CBTT and SWMT (r = 0.117, *p* = 0.203) were not correlated. CBTT and SWMT were positively correlated for backward correct responses (r = 0.350, *p* < 0.01). CBTT and SWMT were positively correlated for backward span (r = 0.359, *p* < 0.01).

#### 3.3.2. Convergent Validity

The convergent validity was analyzed to verify the validity of the CBTT developed in this study. Convergent validity deals with the same concept in theory and is a method of verifying validity through correlation analysis with existing measurement tools that have already been validated. The higher the correlation coefficient, the higher the convergent validity. To this end, a correlation analysis between CBTT and existing standardized tests (K-TMT-e Type B, SMT) was conducted.

##### Correlation Analysis of CBTT and K-TMT-e Type B Performance

To examine the convergent validity of the CBTT, we conducted a correlation analysis with the standardized test, K-TMT-e Type B. The results are presented in Table 4. The backward metrics (correct responses and span) generally showed significant negative correlations with both reaction time and errors on the K-TMT-e Type B, whereas the forward metrics were not significantly correlated. However, forward correct responses showed a significant negative correlation with K-TMT-e Type B errors (r = −0.220, *p* < 0.05).

##### Correlation Analysis of CBTT and SMT Performance

To examine the convergent validity of CBTT, we conducted a correlation analysis with SMT, a standardized test. The results are presented in Table 5. The forward metrics of the CBTT (correct responses and span) showed no significant correlations with SMT reaction time or errors. Among the backward metrics, correct responses showed significant negative correlations with SMT reaction time and errors, whereas span did not show a significant correlation.

#### 3.3.3. Sensitivity Analysis

A sensitivity analysis was conducted to examine the robustness of the results with respect to outlier exclusion. Comparing correlation coefficients between the sample including outliers (*N* = 132) and the final sample excluding outliers (*N* = 120), the backward correct responses and backward span maintained significant correlations in most cases regardless of outlier inclusion, whereas some correlations involving the forward metrics that were significant when outliers were included became non-significant after outlier exclusion.

## 4. Discussion

The purpose of this study was to develop and validate a mobile Corsi-Block Tapping Test as a tool for assessing visuospatial working memory among community-dwelling older adults. Participants in this study were community-dwelling older adults, who may face difficulty in regularly visiting hospitals or undergoing cognitive assessments due to physical limitations or a lack of healthcare infrastructure in their region of residence [35]. Given that smartphones are relatively accessible devices, mobile-based cognitive assessments hold potential as an alternative evaluation method that could help address these limitations. Furthermore, mobile assessments can be administered repeatedly without constraints of time or location, suggesting the possibility that they may serve as a reference tool for individuals to monitor changes in their own cognitive function in the future.

The main findings of this study are as follows.

First, to assess the reliability of the CBTT, Cronbach’s α, an metrics of internal consistency, was calculated. In this study, the internal consistency of the CBTT was α = 0.767, indicating an acceptable level. However, due to the characteristics of the test system used in this study, the CBTT was designed to record only four summary metrics (forward correct responses, forward span, backward correct responses, and backward span) rather than individual trials; accordingly, Cronbach’s α was calculated using these summary metrics as items rather than individual trial data. This differs from conventional internal consistency analysis [36], and therefore warrants some caution in interpretation, but overall suggests that the metrics of the CBTT possess a reasonably consistent internal structure.

Second, to examine the concurrent validity of the CBTT, a correlation analysis was conducted with the SWMT. The results showed a weak positive correlation for forward correct responses, and moderate positive correlations for both backward correct responses and backward span, whereas forward span showed no significant correlation. The finding that three of the four CBTT metrics showed significant positive correlations at the small-to-medium level partially supports the notion that the CBTT measures a construct similar to that assessed by the SWMT, an already validated measure. In particular, the relatively larger correlations observed for the backward metrics compared to the forward metrics may be attributable to the fact that the backward task requires not only the storage of information but also the processing involved in reconstructing its order, thereby more closely reflecting the characteristics of working memory [6]. On the other hand, the lack of a significant correlation for forward span may be related to the relatively lower stability of the span metric, which can be overestimated by chance rather than reflecting actual ability [12].

Third, to examine the convergent validity of the CBTT, a correlation analysis was conducted with the K-TMT-e Type B. The results showed that CBTT forward correct responses and span were not significantly correlated with either reaction time or errors on the K-TMT-e Type B. In contrast, CBTT backward correct responses showed weak-to-moderate significant negative correlations with both K-TMT-e Type B reaction time and errors, and CBTT backward span showed a moderate significant negative correlation with K-TMT-e Type B reaction time. CBTT backward span was not significantly correlated with K-TMT-e Type B errors. These findings are partially consistent with previous studies that examined validity through correlation analyses between the TMT and the Corsi block-tapping test. According to Baykara et al. [37], a tablet-based Corsi block-tapping task and TMT-B were negatively correlated. The TMT subtests, Parts A and B, are known to assess different cognitive domains: Part A is largely associated with simple visual search and attention, whereas Part B, which requires alternating between numbers and letters while also maintaining sequential order, tends to be more closely associated with visuospatial ability and working memory [38,39]. The finding that the backward metrics of the CBTT, but not the forward metrics, showed significant correlations with the K-TMT-e Type B in the present study can be interpreted as reflecting the fact that the backward task of the CBTT, like the K-TMT-e Type B, shares the core characteristic of working memory in requiring simultaneous storage and manipulation of information. These findings suggest that the CBTT, particularly its backward metrics, may be a sensitive indicator of visuospatial working memory ability.

Fourth, to examine the convergent validity of the CBTT, a correlation analysis was conducted with the SMT. The results showed that CBTT backward correct responses had a weak but significant negative correlation with both SMT reaction time and errors. However, CBTT forward correct responses, forward span, and backward span were not significantly correlated with SMT reaction time or errors. The SMT requires additional executive functions, such as real-time route planning and foresight, in addition to visuoconstructional ability [33], which differs somewhat from the task characteristics of the CBTT, which involves storing and reproducing static positional and sequential information. This difference is thought to explain why the CBTT showed a relatively more limited association with the SMT compared to its correlations with the SWMT and K-TMT-e Type B reported above. Nevertheless, consistent with the results obtained for the SWMT and K-TMT-e Type B, the finding that CBTT backward correct responses was significantly correlated with the SMT provides consistent support for this metric being the most stable indicator underpinning the validity of the CBTT.

The present study has the following limitations.

First, the sample of this study has a limitation in that it is biased with respect to age, sex, and region. Approximately 83% of the sample fell within the 65–69 age range; older adults are generally defined as individuals aged 65 years or older, and increasing age is known to be associated with a greater risk of cognitive decline [40,41]. In addition, male participants outnumbered female participants by more than threefold. Previous studies have reported that men tend to perform somewhat better than women on visuospatial tasks, and although the magnitude of this sex difference has decreased in recent years, it remains significant [42]. Given this age and sex imbalance, it cannot be ruled out that the sample of this study included a relatively larger proportion of individuals with well-preserved cognitive function, which may have resulted in task performance levels higher than the average for the broader older adult population. Furthermore, participants in this study were recruited from a single region. Smartphone penetration and familiarity may vary by region [43]; however, since information on participants’ familiarity with smartphone use was not separately collected in this study, the potential influence of such regional differences on the results could not be determined. Therefore, whether the findings of this study can be equally applied to a broader population warrants confirmation through future research.

Second, this study did not evaluate test–retest reliability. In addition, because the study was conducted in a controlled testing environment using a single smartphone model, it remains unclear whether the same level of reliability would be maintained when the test is administered in real-world settings using a variety of devices. Therefore, future research should verify test–retest reliability through repeated measurements and examine the stability of the CBTT across diverse environmental and device conditions.

Third, this study did not examine the validity of the CBTT against the traditional paper-and-pencil Corsi block-tapping task. As correlational analysis with an already validated existing measure is a representative method for establishing the validity of a newly developed test [13,44], a correlation analysis with the traditional Corsi block-tapping task may also have been necessary in this study. However, the CBTT developed in this study presents stimuli in the form of color changes in blocks on a two-dimensional screen, whereas the traditional paper-and-pencil version involves the examiner physically tapping blocks with a finger in three-dimensional space. According to previous research, observing the movement of the examiner’s finger as it moves to the next block during the paper-and-pencil version may serve as an additional cue for positional memory [15,45]. Accordingly, to minimize the confounding variable arising from this difference in stimulus presentation, the present study examined the validity of the CBTT through a correlation analysis with a computerized Corsi block-tapping test (SWMT) that shares the same stimulus presentation format as the CBTT.

Fourth, the CBTT developed in this study did not establish norms stratified by age and education level. As a result, no reference standard was available for comparing individual scores, which presents a limitation in that the CBTT alone cannot be used to objectively determine the presence of cognitive impairment. Therefore, at the present stage, it would be more appropriate to consider the CBTT as a research tool that has established preliminary evidence for measuring visuospatial working memory, rather than as a tool for clinical diagnosis or screening purposes. If future research were to establish age- and education-stratified norms using a large-scale sample, this would allow for a more precise interpretation of individual performance and enable the CBTT to be utilized as a tool with clinical utility.

Despite these limitations, this study is significant for the following reasons.

First, this study developed a mobile version of the task while carefully considering the psychometric properties originally intended to be measured by the Corsi block-tapping test. The stimulus presentation conditions of the CBTT followed established procedures that have been widely used and validated in clinical settings. The CBTT begins with a sequence of two blocks in the first trial, and the inter-stimulus interval was set at 1000 ms, in an effort to comply with the standard stimulus presentation criteria of the traditional Corsi block-tapping task. In this respect, this study is significant in that it represents an attempt to develop the task while carefully reflecting the psychometric properties of the existing measure.

Second, this study is significant in that it represents one of the initial attempts to develop and validate a Korean mobile-based Corsi block-tapping test. Smartphones are widely adopted among adults in South Korea, with a usage rate of 98% among Korean adults and exceeding 90% among those aged 70 years and older (https://www.gallup.co.kr/gallupdb/reportContent.asp?seqNo=1497&utm_source=chatgpt.com, accessed on 7 August 2026). Unlike computer-based assessments, which require a separate monitor and mouse, mobile-based assessments have the advantage of being administered using only a smartphone. This characteristic has the potential to contribute to creating an environment in which older adults can more readily access cognitive function assessments.

## 5. Conclusions

This study aimed to develop a Korean mobile-based Corsi Block-Tapping Test (CBTT) and to examine its reliability and validity among community-dwelling older adults. The internal consistency of the CBTT was acceptable (α = 0.767), and with respect to validity, backward correct responses in particular showed consistent significant correlations with several criterion measures, suggesting that the CBTT demonstrates preliminary validity as a measure of visuospatial working memory. However, as noted above, given limitations such as sample bias, the absence of test–retest reliability assessment, and the lack of established norms, the CBTT should currently be regarded as a preliminary research tool rather than a standalone diagnostic or screening instrument. Furthermore, given that clinical practice often relies on a battery composed of two or more tests rather than a single test for more accurate diagnosis [46], it is expected that the CBTT’s utility in clinical and community screening settings would be further enhanced if it were used in combination with other cognitive assessments in the future.

## Figures and Tables

**Table 1:** Demographic characteristics of study participants (*N* = 120).

Attributes	Separation	Frequency (%) or Mean (SD)
Gender	male	89 (74.2%)
	female	31 (25.8%)
Age, M (SD)		69.29 (4.33)
Age	65 to 69	83 (69.2%)
	70 to 74	21 (17.5%)
	75 to 79	10 (8.3%)
	80 to 84	6 (5%)
Education	1 to 6	5 (4.2%)
	7 to 9	16 (13.3%)
	10 to 12	58 (48.3%)
	13+	41 (34.2)
Measurement Tools	K-MMSE-2:SV (T score)	50.98 (7.74)
	CDR	0.325 (0.24)
	SGDS	1.27 (1.79)

**Table 2:** Descriptive statistics of the main variables (*N* = 120).

Variables	Mean (M)	Standard Deviation (SD)
CBTT Forward Correct Responses	7.17	1.96
CBTT Forward Span	5.16	1.17
CBTT Backward Correct Responses	6.44	1.90
CBTT Backward Span	4.88	1.08
SWMT Forward Correct Responses	4.61	1.34
SWMT Forward Span	6.17	2.21
SWMT Backward Correct Responses	4.94	1.62
SWMT Backward Span	6.52	2.56
K-TMT-e Type B Reaction Time	44.87	37.70
K-TMT-e Type B Error	0.85	1.71
SMT Response Time	31.07	12.30
SMT Total Error	0.38	0.85

Note. CBTT = Mobile Corsi Block Tapping Test. SWMT = Spatial Working Memory Test of the Comprehensive Attention Test. K-TMT-e Type B = The Korean Trail Making Test for the Elderly Type B. SMT = Snellgrove Maze Test.

**Table 3:** Correlation between CBTT and SWMT (N = 120).

	1	2	3	4	5	6	7	8
1. CBTT Forward Correct Responses	1							
2. CBTT Forward Span	0.832 **	1						
3. CBTT Backward Correct Responses	0.401 **	0.344 **	1					
4. CBTT Backward Span	0.296 **	0.258 **	0.829 **	1				
5. SWMT Forward Correct Responses	0.258 **	0.114	0.356 **	0.343 **	1			
6. SWMT Forward Span	0.217 *	0.117	0.297 **	0.281 **	0.874 **	1		
7. SWMT Backward Correct Responses	0.188 *	0.182 *	0.350 **	0.309 **	0.444 **	0.419 **	1	
8. SWMT Backward Span	0.245 **	0.233 *	0.434 **	0.359 **	0.486 **	0.449 **	0.917 **	1

Note. CBTT = Mobile Corsi Block Tapping Test. SWMT = Spatial Working Memory Test of the Comprehensive Attention Test. * *p* < 0.05, ** *p* < 0.01.

**Table 4:** Correlations between CBTT and K-TMT-e Type B performance (N = 120).

	1	2	3	4	5	6
1. CBTT Forward Correct Responses	1					
2. CBTT Forward Span	0.832 **	1				
3. CBTT Backward Correct Responses	0.401 **	0.344 **	1			
4. CBTT Backward Span	0.296 **	0.258 **	0.829 **	1		
5. K-TMT-e Type B Reaction Time	−0.124	−0.125	−0.291 **	−0.272 **	1	
6. K-TMT-e Type B Error	−0.220 *	−0.171	−0.331 **	−0.166	0.603 **	1

Note. CBTT = Mobile Corsi Block Tapping Test. K-TMT-e Type B = The Korean Trail Making Test for the Elderly Type B. * *p* < 0.05, ** *p* < 0.01.

**Table 5:** Correlation between CBTT and SMT performance (N = 120).

	1	2	3	4	5	6
1. CBTT Forward Correct Responses	1					
2. CBTT Forward Span	0.832 **	1				
3. CBTT Backward Correct Responses	0.401 **	0.344 **	1			
4. CBTT Backward Span	0.296 **	0.258 **	0.829 **	1		
5. SMT Reaction Time	−0.066	−0.055	−0.187 *	−0.138	1	
6. SMT Total Error	−0.031	−0.087	−0.202 *	−0.068	0.079	1

Note. CBTT = Mobile Corsi Block Tapping Test. SMT = Snellgrove Maze Test. * *p* < 0.05, ** *p* < 0.01.

## Data Availability

The data presented in this study are available on request from the corresponding author. The data are not publicly available due to privacy and ethical restrictions related to research participants.

## References

[1] Fu X., Ji S., Li J., Chen T., Liu X., Kuang W. (2025). Cognitive impairment and dependency in activities of daily living: A cross-lagged analysis. Front. Aging Neurosci..

[2] Parsey C.M., Schmitter-Edgecombe M. (2013). Applications of technology in neuropsychological assessment. Clin. Neuropsychol..

[3] Baddeley A. (2003). Working memory: Looking back and looking forward. Nat. Rev. Neurosci..

[4] Shin E., Brumback-Peltz C., Gordon B.A., McAuley E., Gratton G., Fabiani M. (2012). The effects of aging and physical fitness on working memory capacity. Korean J. Cogn. Biol. Psychol..

[5] Bopp K.L., Verhaeghen P. (2005). Aging and verbal memory span: A meta-analysis. J. Gerontol. Ser. B Psychol. Sci. Soc. Sci..

[6] Baddeley A. (2020). Working memory. Memory.

[7] Wang E.H., Lai F.H., Leung W.M., Shiu T.Y., Wong H., Tao Y., Zhao X., Zhang T.Y., Yee B.K. (2025). Assessing rapid spatial working memory in community-living older adults in a virtual adaptation of the rodent water maze paradigm. Behav. Brain Res..

[8] Muffato V., Borella E., Pazzaglia F., Meneghetti C. (2022). Orientation experiences and navigation aid use: A self-report lifespan study on the role of age and visuospatial factors. Int. J. Environ. Res. Public Health.

[9] Corsi P.M. (1973). Human memory and the medial temporal region of the brain. Diss. Abstr. Int..

[10] Milner B. (1971). Interhemispheric differences in the localization of psychological processes in man. Br. Med. Bull..

[11] Trojano L., Grossi D. (1994). A critical review of mental imagery defects. Brain Cogn..

[12] Kessels R.P., Van Zandvoort M.J., Postma A., Kappelle L.J., De Haan E.H. (2000). The Corsi block-tapping task: Stand-ardization and normative data. Appl. Neuropsychol..

[13] Brunetti R., Del Gatto C., Delogu F. (2014). eCorsi: Implementation and testing of the Corsi block-tapping task for digital tablets. Front. Psychol..

[14] Farrell Pagulayan K., Busch R.M., Medina K.L., Bartok J.A., Krikorian R. (2006). Developmental normative data for the Corsi Block-tapping task. J. Clin. Exp. Neuropsychol..

[15] Claessen M.H., Van Der Ham I.J., Van Zandvoort M.J. (2015). Computerization of the standard corsi block-tapping task affects its underlying cognitive concepts: A pilot study. Appl. Neuropsychol. Adult.

[16] Siddi S., Preti A., Lara E., Brébion G., Vila R., Iglesias M., Cuevas-Esteban J., López-Carrilero Raquel R., Butjosa A., Haro J.M. (2020). Comparison of the touch-screen and traditional versions of the Corsi block-tapping test in patients with psychosis and healthy controls. BMC Psychiatry.

[17] Wilson S.A., Byrne P., Rodgers S.E., Maden M. (2022). A systematic review of smartphone and tablet use by older adults with and without cognitive impairment. Innov. Aging.

[18] Demeyere N., Haupt M., Webb S.S., Strobel L., Milosevich E.T., Moore M.J., Wright H., Finke K., Duta M.D. (2021). Introducing the tablet-based Oxford Cognitive Screen-Plus (OCS-Plus) as an assessment tool for subtle cognitive impairments. Sci. Rep..

[19] Terwee C.B., Mokkink L.B., Knol D.L., Ostelo R.W., Bouter L.M., de Vet H.C. (2012). Rating the methodological quality in systematic reviews of studies on measurement properties: A scoring system for the COSMIN checklist. Qual. Life Res..

[20] Tukey J.W. (1977). Exploratory Data Analysis.

[21] Folstein M., Folstein S., McHugh P. (1975). “Mini-mental state”. A practical method for grading the cognitive state of patients for the clinician. J. Psychiatr. Res..

[22] Kang Y., Jang S., Kim S. (2020). K-MMSE-2 Korean Version of the Simplified Mental State Test.

[23] Choi S.-H., Na D.-R., Lee B.-H., Ham D.-S., Jung H., Yoon S.-J., Yoo K.-H., Ha C.-G., Han I.-W. (2001). Estimating the Validity of the Korean Version of Expanded Clinical Dementia Rating (CDR) Scale. J. Korean Neurol. Assoc..

[24] Yesavage J.A., Brink T.L., Rose T.L., Adey M. (1983). The Geriatric Depression Rating Scale: Comparison with other self-report and psychiatric rating scales. Assess. Geriatr. Psychopharmacol..

[25] Sheikh J.I., Yesavage J.A. (1986). Geriatric Depression Scale (GDS): Recent evidence and development of a shorter version. Clin. Gerontol. J. Aging Ment. Health.

[26] Cho M.J., Bae J.N., Suh G.H., Hahm B.J., Kim J.K., Lee D.W., Kang M.H. (1999). Validation of geriatric depression scale, Korean version (GDS) in the assessment of DSM-III-R major depression. J. Korean Neuropsychiatr. Assoc..

[27] Ki B.-S. (1996). A Preliminary Study for the Standardization of Geriatric Depression Scale Short Form-Korea Version. J. Korean Neuropsychiatr. Assoc..

[28] Ham H.K., Park Y.B. (2011). Mobile application compatibility test system design for android fragmentation. International Conference on Advanced Software Engineering and Its Applications.

[29] Jackson W. (2013). Android DIP: Device-Independent Pixel Graphics Design. Pro Android Graphics.

[30] Orsini A., Pasquadibisceglie M., Picone L., Tortora R. (2001). Factors which influence the difficulty of the spatial path in Corsi’s block-tapping test. Percept. Mot. Ski..

[31] Yoo H.I. (2009). Standardization of the Comprehensive Attention Test for the Korean children and adolescents. J. Korean Acad. Child Adolesc. Psychiatry.

[32] Park M.S., Choi J.Y. (2003). Normative study of the modified Trail Making Test (TMT) for Korean older adults. Korean J. Clin. Psychol..

[33] Snellgrove C.A. (2005). Cognitive Screening for the Safe Driving Competence of Older People with Mild Cognitive Impairment or Early Dementia.

[34] Nunnally J.C. (1978). Psychometric Theory.

[35] Miller J.B., Wong C.G., Caldwell J.Z., Rodrigues J., Pudumjee S., John S.E., Ritter A. (2023). Cognitive aging in rural communities: Preliminary memory characterization of a community cohort from Southern Nevada. Front. Dement..

[36] Cronbach L.J. (1951). Coefficient alpha and the internal structure of tests. Psychometrika.

[37] Baykara E., Kuhn C., Linz N., Tröger J., Karbach J. (2022). Validation of a digital, tablet-based version of the Trail Making Test in the∆ elta platform. Eur. J. Neurosci..

[38] Sánchez-Cubillo I., Periáñez J.A., Adrover-Roig D., Rodríguez-Sánchez J.M., Ríos-Lago M., Tirapu J.E.E.A., Barceló F. (2009). Construct validity of the Trail Making Test: Role of task-switching, working memory, inhibition/interference control, and visuomotor abilities. J. Int. Neuropsychol. Soc..

[39] Libon D.J., Swenson R., Tobyne S., Jannati A., Schulman D., Price C.C., Lamar M., Pascual-Leone A. (2024). Dysexecutive difficulty and subtle everyday functional disabilities: The digital Trail Making Test. Front. Neurol..

[40] James D.L., Larkey L.K., Maxfield M., Han S., Ofori E., Mohr A.E., Hawley N.A., Alperin K., Ahlich E., Vance D.E. (2025). Prolonged nightly fasting in older adults with memory decline: A single-group pilot study exploring changes in cognitive function and cardiometabolic risk factors. J. Clin. Transl. Sci..

[41] Shatenstein B., Barberger-Gateau P. (2015). Prevention of age-related cognitive decline: Which strategies, when, and forWhom?. J. Alzheimer’s Dis..

[42] Voyer D., Voyer S.D., Saint-Aubin J. (2017). Sex differences in visual-spatial working memory: A meta-analysis. Psychon. Bull. Rev..

[43] Wang X., Zhao Y. (2024). Does smartphone use affect attitudes toward aging among older adults in rural areas? An empirical analysis using data from the Chinese longitudinal aging social survey. Behav. Sci..

[44] Woods D.L., Wyma J.M., Herron T.J., Yund E.W. (2016). An improved spatial span test of visuospatial memory. Memory.

[45] Brass M., Bekkering H., Prinz W. (2001). Movement observation affects movement execution in a simple response task. Acta Psychol..

[46] Russell E.W., Russell S.L.K., Hill B.D. (2005). The fundamental psychometric status of neuropsychological batteries. Arch. Clin. Neuropsychol..

